# Massachusetts Dental Society

**Published:** 1903-02

**Authors:** 


### MASSACHUSETTS DENTAL SOCIETY.

(Afternoon session, continued from page 50.)

*President Faxon.*—We will now ask Dr. Edward C. Kirk, of Philadelphia, to give his lecture on "Saliva," with lantern exhibits.

[This lecture is not in the report, but as it has been repeated several times, Dr. Kirk prefers to refer the reader to his various papers on this subject. See page 229, Vol. XXIII., INTERNATIONAL DENTAL JOURNAL.—ED.]

DISCUSSION.

*Dr. Samuel A. Hopkins.*—Mr. Chairman, ladies, and gentlemen, I know that you might easily have chosen some one more capable of discussing this brilliant lecture of Dr. Kirk's, but I am sure that you could not have chosen any one who was more interested or more appreciative.

As I sat listening attentively to the paper and as I followed closely the lucid explanations of the methods employed in these investigations, it pleased me to think that this afternoon and this talk might mark a new epoch in dentistry, and that this Society might have the honor of being the agent through which this epoch-making knowledge should be given to the world.

Dr. Kirk has been too long a leader in dental societies and in dental literature to desire the flattery which might be pleasing and encouraging to a younger man. He has passed the age where personal advancement is preferred to scientific accuracy.

In this world the one thing supremely worth having is the opportunity, coupled with the capacity, to do a piece of work the doing of which shall be of vital importance to mankind. It has been Dr. Kirk's privilege to fill this proud position.

In 1895 Dr. Michaels, who deserves the gratitude of the profession for suggesting to the world this line of investigation, published a paper on "abrasions." Later he published another paper which was much clearer and went much farther than the first one. I read the first paper with much interest but I fear with but little understanding, and I believed myself most ignorant because I could not in the least understand what Dr. Michaels was aiming at. I found that this was the attitude of many other members of the profession towards this original announcement of Dr. Michaels's work. He seemed to me to be in the position of a man who has a valuable possession in some far-away country. He knows that it is there and he knows that it is valuable, but so far from being able to describe it to the world, he hardly knows what it looks like himself and scarcely knows how to get to it.

Dr. Kirk has cleared up this mystery and has given us an exceedingly clear insight into this new department of scientific research, and has to some degree shown us the practical application of it. So far as it relates to general disease, this knowledge is bound to mark an advance in diagnosis and throw new light on general conditions that are at present but little understood. I am hopeful that the mystery surrounding the subject of abrasions will be cleared up, and that as a result of these investigations we shall be able to cope successfully with that distressing condition. So far as it bears upon our knowledge of caries (if I may be permitted to express myself in opposition to Dr. Michaels), I think we have not made any material advance by reason of his work. Further advances cannot be made without taking into consideration the work of bacteria and the products of bacterial activity. Physiological chemistry is also a science which must not be ignored in the study of mouth secretions and their effect upon the teeth. A knowledge of those wonderful energy transforming substances, the enzymes, the development of the wonderful ionic theory by which substances in infinite solution have an action which is denied them in their higher states and have also an affinity for the poles of a battery,—these and a further study of electrical action will have much to do with improvement in our skill in dealing with mouth conditions. What we need most at the present time is to have our knowledge catalogued and divided in such a way as to bring it within the dentist's power to use it. There is a great deal of general knowledge which never has a practical application in dental practice, because it is obscured by cumbersome theories and mystifying technicalities. This new knowledge which Dr. Kirk has brought to our notice may not be absolutely complete, but it certainly opens up the greatest possibilities for dental advancement.

The last perfect specimen of a locomotive could never have been built had not Stephenson built his smaller one; and while it is true that our knowledge of the oral fluids is by no means perfect, we may congratulate ourselves that such a splendid beginning has been made and that the future gives such brilliant promise.

*Dr. James Truman,* Philadelphia, Pa.—Mr. Chairman, I do not know that I have anything to add by way of discussion of this paper, for I have necessarily been almost daily in contact with Dr. Kirk's work, and have realized equally with him the importance of it. I regret, however, that the specimens are not plainer. It seems to me they have deteriorated. They do not present the marked characteristics that I have observed in them previously under the microscope. However, they are important; but the great point to me in this investigation is the endeavor to solve the problem of what constitutes erosion and wherein it differs from abrasion. Our profession has been for the last fifty years endeavoring to understand these two pathological conditions. We have had a great deal of theory, but nothing positive. Now, some of these experiments, which he did not allude to here, seem to demonstrate very conclusively that the action of the acid in the mouth, as he has described, produces erosion upon the labial and buccal surfaces of the teeth. He has also demonstrated another point,—that abrasions in the majority of cases are the result of acid action. This has usually been considered to be the result of attrition alone. I think you all know that this cannot be true, but that it is by the action of a combination of acid and attrition. I have one criticism to make in regard to my friend's lecture, and that is where he makes use of the term "saliva." Now, I do not believe he has analyzed saliva. I think we are making a mistake in the way of terms, and should always in my judgment call this the oral fluid. Saliva cannot be secured except by special effort. I have great faith that we will know more in the future from Dr. Michaels's and Dr. Kirk's work. We practically knew nothing of the oral fluid prior to these investigations, and to me they are extremely valuable.

*President Faxon.*—Dr. Truman has given us great encouragement in one remark,—that is, that we are going to know more. I will call upon Dr. Flanagan to favor us.

*Dr. A. J. Flanagan.*—Mr. President, members, and friends, the president of this Society this morning, when he was disappointed in not having a discussion from the gentlemen who disappointed our Society, and knowing that I had been in the private laboratory of Dr. Kirk for an hour and a half, has taken the liberty of calling upon me. He also made the statement this afternoon that this paper was something by a scientific man and of a scientific nature. Now, as I have sat here this afternoon I said to myself I will get away from the scientific and come down to the status of the every-day dentist. That is, I will speak from the practical stand-point in a very few words. If you will go back in the history of dentistry, you will find that Miller is credited as the discoverer of caries of the teeth, and hence, notwithstanding the fact that many others before him had brought out certain truths along the line of and leading up to the discovery, yet the fact remains that Miller was the man that gave us the one crowning truth. If any of the gentlemen present have read the various thoughts of Dr. Michaels as presented before the International Congress, let him remember that there was very little information of a practical nature in that article that could be grasped from the stand-point of the English-speaking practitioner; but when Dr. Kirk gave a short paper sometime ago on the work of Dr. Michaels, he brought down to a practical stand-point in a very few words the very fundamental thoughts which we need in dentistry. If I might be permitted I would say that I consider that Dr. Michaels was soaring in space when presenting his paper on saliva study, and that Dr. Kirk has brought the telling essential part of his work down to us where we can see it clearly. The one hour and a half I spent in Dr. Kirk's laboratory enabled me to grasp in a few moments just what Dr. Michaels was laboring for, and I am still greater impressed by the fine work Dr. Kirk has thrown on the screen this afternoon. There is one great fact we can now take home, and that is that there is a condition of normal saliva and another of abnormal saliva. Dr. Kirk has demonstrated this without doubt. Then, when we have in the future a puzzling condition in a patient's mouth, showing an abnormal condition of the saliva, may not this abnormality demonstrated so beautifully by Dr. Kirk this afternoon be the key to the treatment? We hear of many practitioners denying the dentist's ability in the pathological and microscopical laboratory, claiming as the criterion the physicians ability along these lines. Let us look into this. How many physicians make the minute examinations of sputum, urine, etc.? Very few, indeed, for the specimens are passed over to the pathologist and microscopist for examination, and the treatment frequently depends on this specialist's findings. Our calling is coming to that stage where we will have to divide into more specialties, and the time is not far distant when we will turn over to the specialist specimens of saliva, urine, etc., for the scientific treatment of certain dental lesions. Dr. Kirk is certainly to be credited as the man who has gathered many ideas along the line of saliva study, and has shown us this day practical deductions. I say practical, because practical, to me, means logical deductions of the scientific to a useful end.

*President Faxon.*—When a person makes remark to a number of people, it is interpreted in different ways according to the person who receives it. As I sat here I saw a person make a movement, and I am going to take advantage of the interpretation and ask him to speak. I will, therefore, ask Dr. Andrews to say a few words.

*Dr. R. R. Andrews,* Cambridge, Mass.—I do not understand the subject, although I appreciate and admire what has been shown. If we can, by the examination of the saliva, determine these things, it is going to be of great value. It is possible, Dr. Michaels tells us, to tell by the chemical analysis of the saliva whether a person has decayed teeth or not. If we can by proper nutrition bring about a normal condition of the saliva, we shall have accomplished a great deal. It will be a wonderful help if by the examination of the saliva of the sick we can determine the disease and by this means get at the truth. It seems to me to be a great discovery. I have not studied the subject. I have never made anything but the very simplest analysis of saliva, and I do not feel that I am equal to this occasion. I can admire Dr. Kirk's work, and see in the future great advances from this branch of investigation.

*President Faxon.*—Now I am going to ask if there is not some one else present who would like to say a few words upon this subject? If there is, we would be very glad to listen to him. The floor is open to any one here. Dr. Stockwell's name has been mentioned.

*Dr. Chester T. Stockwell,* Springfield, Mass.—I am both glad and sorry to be called upon. I am glad to join with you all in expressions of appreciation and gratitude for what Dr. Kirk has so clearly exhibited and demonstrated here. I am sorry, for I certainly cannot undertake at the present time to discuss the paper. It is for us to think about it, to study the matter, rather than to talk much about it. The subject is almost new to me, but it impresses me as a most important one. And so I heartily agree with Dr. Andrews when he says that we have here opened up to us a field of wonderful interest and promise, and one that is sure to result in great good, not only to the profession but to humanity at large. We can all see at once, I think, that this line of research is bound to be of great service in diagnosis, and no less so in the realm of prophylaxis.

There is another reason why I am especially glad for this paper. It indicates a new scientific era in the world of dentistry and a return from what has for some time seemed to me a too exclusive devotion to the purely mechanical or art side of our profession. This is important, it is true, but we are to depend, I think, upon the development of the scientific aspect of dentistry if we are to maintain the claim of being a profession rather than a class of artisans. In this connection, therefore, I have a suggestion to offer. Let all our district societies take up this matter and focus upon it and around it their fall and winter's work. In this way we may best conserve our own interests, and likewise best manifest our appreciation of Dr. Kirk's magnificent pioneer work. We may follow safely where he so well leads.

Adjourned till 6.30 P.M.

### *Evening Session.*

At 6.30 o'clock a banquet was enjoyed by one hundred and fifty members and friends of the Society. After this was disposed of the president addressed the members as follows:

*President Faxon.* — The subject selected for this evening's meeting seems a big one for an after-dinner topic, but it is considered an advantage to make our banquets educational and instructive by calling together some of the best men at times to discuss different subjects, different matters of general interest, as well as to make them a pleasure by the presence of the ladies and good music. Last year "Dental Education" was ably discussed, and I am certain that those present were well informed in regard to the advance that is being made in that direction, and next in line of importance to us is dental legislation. It is wisely said that we have laws enough now, both good and bad; let us be careful not to add to the bad ones. It is also said that all legislation to-day prescribing and controlling the qualifications and standard of dentistry has been effected through the influence of the State societies. The committee in choosing the first speaker of the evening have shown good judgment in choosing one who has appeared before the State Legislature of Massachusetts in behalf of the State Dental Society and in the interests of dentists generally throughout the State. It is with feelings of great pride that I ask you to listen to the Hon. James A. McGeough, of Boston.

MR. PRESIDENT, LADIES, AND GENTLEMEN,—Some of us here to-night I know are not members of the profession or of this honored Society, but from the way in which we all seem to have enjoyed this bounteous feast just now before us, I think I may fairly claim that we are all pretty good dentists in a way, and those of us not members are, for the time being, at least, closely affiliated with the Society,—a kind of honorary members as it were,—and therefore, Mr. President, to be sociable, may I not be permitted to include all and say, Ladies and Gentlemen of the Massachusetts Dental Society.

As our president was closing his remarks just now, and I realized that he was about to call my name, I began to feel very much like the man who had prepared his speeches very carefully in advance, but when the time came for delivery had almost forgotten the words. However, the kind introduction on the part of our president and the very generous applause accorded me at the outset give me assurance that I am in the house of my friends, and that in filling out my allotted part of this interesting programme the blunders and shortcomings I may be guilty of will all be kindly overlooked or forgiven.

At these annual meetings of the Society I understand that, aside from the social features, it is the custom to introduce some subject for discussion that may tend to an advancement of the science and the good of the profession, and on this occasion it is, as announced, "The Law in Relation to the Practice of Dentistry."

During the past few years this subject has engaged the attention of the courts from time to time and has become a matter of considerable importance not only to those seeking admission to the profession, but to those already in, as well as to the public at large. It is also important because there seems to be some difference of opinion as to the form and fairness of the existing law, and I have been requested to say something about this subject, because it is a subject about which I suppose I am presumed to know. I appreciate the honor, and, without intending to trespass on your patience long, I trust I may be able to render a little assistance in solving some of the difficulties and questions involved.

Now, I am not going to tell you what I know about dentistry, although it would not take me long; nor am I going to tell you what I do not know about dentistry, because, while that might be very interesting in many respects, it would necessitate a waste of altogether too much time, and therefore I hope to be excused if I studiously avoid that interesting subject altogether except in a very general way. Fortunately for me, however, it is not necessary to be much of a dentist in order to be able to talk about the law; and law of any kind is, I also beg leave to say, rather a serious subject for a festive occasion, and if I become tiresome I pray your indulgence. I must endeavor, of course, to keep close to the subject announced for the evening.

In the first place, law is not a thing to be desired for itself exactly. Law always implies a restriction of liberty. It denotes the existence or possibility of some public wrong. It is generally associated in the mind with fines, penalties, and imprisonment, and some may naturally ask the question, Why should there be any law in relation to the practice of dentistry? What is the reason for it? Where is the necessity?

This is an important question. It lies at the foundation of every law. A correct knowledge of the origin, the necessity, and purpose of a law goes far to a proper construction and a better understanding of the law itself, and therefore, instead of a tiresome recital of the many controversies and mooted questions that have arisen from time to time in and out of court, I think it better and more satisfactory if we give this particular phase of the subject a somewhat extended consideration: Why should there be any law in relation to the practice of dentistry?

Briefly stated, the answer to this question is found in the present prosperous condition of the profession, the large and increasing number of practitioners, and in the nature and growing popularity of the science itself as affecting the public at large; and to this latter consideration more particularly I wish to call attention.

Dentists or no dentists, we all know enough to know that it is a good thing to have good teeth, especially on occasions like this, for instance; and in order to have good teeth we must preserve them; and we all know enough to know that when by accident or decay we unfortunately lose our teeth, it is of the highest importance that we have their places filled by the very best substitutes which the skill and ingenuity of the profession can supply.

Doctors tell us, and I am not so sure but what it is now a matter of very general, if not universal, knowledge, that the physical well-being and good health of the body depend largely upon the care and preservation of the teeth. I have no doubt that neglected old roots and decayed teeth are often responsible in a great measure for many of the other ills that flesh is heir to. So that in many ways and for various reasons we know it is a good thing to have good teeth. Good teeth are essential to a pleasant and a clear articulation. We do not speak or sing with our teeth exactly, but what kind of a speech could a Daniel Webster make, and what kind of a song could even a Patti or a Calve sing, without their teeth?

Then there is the æsthetic side of the question, which ought not to be overlooked. Every man should look his best. I fear we men are apt to be a little careless in this respect, but the ladies have no excuse. Somebody has very properly said that it is the duty of ever woman to make herself as beautiful and as attractive as she can, and how can man or woman be attractive without a fairly good set of teeth? Imagine the face of some fair and radiant maiden, with an outline of feature perfect as a Raphael's Madonna; a shock of golden hair, or such as a Titian might envy; a brow white and as smooth as steel; a pair of Vicking blue eyes that flash and shine like buttons on an angel's garment, metaphorically speaking; "the least little delicate aquiline curve in a sensitive nose," said to be a great charm of feature; cheeks aglow with health and beauty, and lips chiselled to perfection and wreathed in happy smiles,—imagine all this, and yet I think it will be at once agreed, without a good set of fairly perfect teeth the picture is incomplete. From every point of view we know it is a good thing to have good teeth.

Besides, dentistry has come to be an attractive and a learned profession. We are told that "the proper study of mankind is man," and while dentistry is more particularly confined to a fraction of the human anatomy, it is none the less interesting and important, and a man only needs to feel the sting of an aching dental nerve for a minute to realize "how fearfully and wonderfully we are made." Formerly no doubt the fear predominated, but the wonder remains. And when you come to think of it, how much of the mystery and wonder of all creation is found in a little tooth! the marvellous origin and growth; the admirable construction and adaptation of parts; the perfection of design; the entire fitness for the end intended; its life, its development, decay, and death, following out and exemplifying in its little round of existence that great and incomprehensible law to which all created material things are subject,—the unchanged and unchanging law of nature, a thorough and practical knowledge of which in its operations and external effect is so essential to the scientific care and preservation of the teeth. From this it will be seen at once that in dentistry, as in all other branches of the medical profession proper, a sound and liberal education is required. Manual dexterity is of course necessary. But a thorough study and practical knowledge of the structure and pathology of the teeth and adjacent parts, the causes of disease and decay, are also essential to the qualifications of a good dentist; and I have no doubt it is this very scientific and studious research in recent years that has helped to elevate the profession to its present high plane of perfection, respectability, and usefulness.

Why should there be any law in relation to the practice of dentistry? A glance at the history of the profession—what it is and what it has been—will help to explain. The contrast is interesting and instructive.

Formerly, and not so very long ago either, the practice of dentistry was anything but attractive. If dentistry was known and practised among the ancient Egyptians in any degree of perfection, as is claimed by some, it certainly became one of the lost arts. As now practised, I understand dentistry is of a very modern date,—fifty years ago; and perhaps some of the older members of the profession to-day may fairly claim to have been at its birth. Prior to that time and for the centuries before, there was little need of any law for the regulation of the practice of dentistry, although they do tell some harrowing tales of the practice in those early days.

If the doctors ever performed any dental operation at all, it was only in case of dire necessity, and the less they had of the business no doubt the better they liked it. In those olden times the practice consisted chiefly in the extracting of fractious and troublesome teeth, and we are told it was main strength on the one side and heroic endurance on the other, as the operation was performed with instruments something like a blacksmith's forceps and a cold chisel. In those happy days it must have been easy for a dentist to collect his bills, for after being dragged about the floor and generally used up, as the stories have it, the patient must have considered that the operator had earned his fee. But sometimes, it is said, things took a different turn. When the suffering patient happened to be a big man with a bad toothache and a worse temper, the operation sometimes assumed the character and proportions of an incipient prize-fight, and as between patient and practitioner it was often a question as to which was the better man.

I am reminded here of the man who came to his work one morning with a black eye and many bruises about his face, and looking generally used up. Seeing his condition, his employer inquired, "Why, Patrick, what happened?" "Oh," said Patrick, in rather an apologetic way, "I was at a wedding last night." "At a wedding!" said his employer; "but how did this happen?" "Well," said Patrick, "it was this way: You see, there were a lot of fine people there putting on a lot of airs, and among them there was one man in particular, with a great deal of white linen and a swallow-tail coat, a-swelling around, and I said to him, said I, 'And who might you be?' and he said to me, 'I'm the best man.' And, begorra, he was."

In those prehistoric days of the profession there was little or no competition. A dentist's office was hard to find. A suffering patient had no difficulty in making a date if he found his dentist; at least there was no crowding or waiting for turns in those days, and seldom, if ever, was there any occasion to invoke the law. Now all this is changed. Dentistry to-day is in comparison a pleasure and a pastime. A dental office is no longer a place of torture, but a "parlor" of luxury and ease, and the throng of patients from day to day attest the popularity and success of the profession. The reason of all this is obvious. We may read it in the signs of the times. Daily from my office window, in great luminous letters of heroic size, so that all who run may read, and a long way off too, if necessary, I am compelled to notice these fascinating specimens: "Big Dental Office," "Teeth extracted without Pain," "Lowest Prices in Boston for Artificial Teeth and all Dental Work," with attractive pictures of the work and specimens of the teeth extracted. What the size of the office has to do with the practice in these days, I don't know. It may be to accommodate the waiting and anxious public. Then, as a part of this same sign, in smaller, but still attractive, letters, I read, "Examinations free," "Bridge-work, $5.00," "Gold crown, $5.00," "Headquarters for artificial teeth, full set, $5.00." On a building front in the next block there used to be a sign, "Full set, $2.50," but that has been recently taken down. I suppose the lucky dentist got rich and retired. But the most interesting of all these alluring signs is this, in small confidential type: "Dr. ———, the *real* painless Dentist." It is so comforting to know that while you are being painlessly operated on, the operator himself is also free from pain.

These, I understand, are but samples of what we now find in many parts of the city of Boston and in every other city and town of importance throughout the country. Even in the street-cars these dental signs now occasionally greet us, and regularly in the daily papers they may be seen in gay and festive columns with "Red Fox Ale," "Divorces procured on the instalment plan," "Clothing on credit; no money down," "Consumption treated, no cure no pay," and cheerful announcements of that kind, with others too numerous and some too unedifying to mention. But I am not discussing the propriety or impropriety of public advertising. I am simply stating some familiar facts, to show the wide-spread and growing popularity of the science itself as affecting the rights and interests of the people.

Then again, dentistry as practised to-day, with all its improved and painless methods, is not free from danger. Grave and serious operations come at times to the dentist's office, and even in minor operations, with the use of anæsthetics, death in the dental chair is not unknown to the profession.

From this condition of things which I have thus endeavored to outline, the reason and necessity for a law regulating the practice of dentistry must be apparent. But to be a little more explicit, it is important to observe in this connection that all good law is and must be for the benefit of the many, not the few. It is a mistake to suppose, as we sometimes hear, that law is for the benefit of any particular number or class of persons. There should be no favoritism under the law. "The greatest good of the greatest number" is the object in view. Incidentally, the individual may and often does reap some benefit or reward, while, on the other hand, some may suffer more or less privation, but the true and only legitimate purpose and object of all law is the welfare and protection of the public. *Salus populi suprema est lex.* This is the test. If it meets this requirement without imposing any unreasonable hardship anywhere, it is a good law, and that this is the true rule a moment's reflection will show.

In primitive society and isolated settlements there is little need of any law. Robinson Crusoe and his man Friday on their lonely island didn't need any law, and in a small Christian community like Acadia, for instance, about which our own Longfellow so beautifully wrote, the ten commandments no doubt would be sufficient. But in the great cities and centres of population an altogether different condition of things exists. The people outside of little circles, more or less extended, although forming the same general community, are practically strangers and unknown to each other, to say nothing of the great floating population in every large city who can have little or no acquaintance of place or people, and yet all these are entitled to and need the protection of the law. Then the difficulties and necessities of life in these days are increased a thousand-fold. The competition in business and trades of all kinds has become very great. There never seems to be money enough to go round, and in the race of life, or rather in the struggle for very existence, the great majority are constantly in danger of being borne to the ground in every direction, and hence the necessity for more specific laws and regulations for their protection and relief.

Now, this rivalry, competition, and strife we find not only in all trades and branches of business, but in the professions as well, and dentistry is no exception to the rule. Incited by the growing commercialism of the times, and attracted by the opportunities which this comparatively new field for money-making affords, it is not to be wondered at if many seek the profession for the money there is in it, while some of those already in, not members of this Society of course, are tempted, I fear, to adopt methods and practices in which the good of the public or honor of the profession has little to do. Accordingly, in Massachusetts, and I understand in most of the States of the Union, there are laws for the regulation of the practice of dentistry.

When you have some plumbing to be done at your house, you want to be assured that the health of your family is not endangered by the faulty construction of an incompetent workman. If you have a prescription to be filled, you want to feel that you may enter any drug-store and have it filled without the fear of being poisoned by the wrong medicine. If a lawyer is to be employed, it is important that he should be a member of the bar. If a doctor or a dentist is wanted, it is only fair to the public that the individual called upon is competent to perform the services required. This is the object of the law, and this the law endeavors to secure.

Now, in the practice of dentistry, as in the practice of any other profession, trade, or calling in which the safety of the public is concerned, there are two ways, and, when you think of it, only two ways, in which the rights and interests of the public are or can be reasonably cared for: First, by admitting to practice those only who are qualified, and secondly, by holding those who are admitted to an exercise of professional skill, due care, and diligence, and to meet these requirements we have two kinds of law,—the statute or written law, and what we call the common or unwritten law.

Let me speak of this second kind of law first, because it is the earlier of the two and in a practical sense perhaps of less importance. I trust I will not offend the strong New England Americanism of any one present when I say that this common law we get not from any legislation of our own, State or national, but we get it, as we get our language, from what may be very properly called in a limited sense the mother-country,—England. It came over in the "Mayflower." It was the guide of the thirteen original Colonies before the Revolution, and to-day it is that great body of jurisprudence, modified and changed by statute more or less to suit the varying conditions of time and place, but still the great body of jurisprudence under which we live. Broad and comprehensive in its scope, it governs and regulates the people in all their rights, duties, and obligations among and to each other,—that of master and servant, employer and employed, doctor and patient, lawyer and client. It includes all those fundamental rules and principles of right and justice recognized in and binding upon all our courts, civil and criminal, and it has been adopted by every State in the Union, except Louisiana alone; and this law we get from England.

Under the provisions of this law every practitioner is bound to a reasonable degree of professional skill and the exercise of due care and diligence, and if by reason of any lack of this professional skill or diligence a patient sustains an injury at his hands, he thereby becomes liable to a suit for damages under the common law.

Of course, politics are not in order here, but I presume patriotism always is, and after this acknowledgment as to the source of our common law I want to say a word by way of explanation and as a kind of set-off, if you will pardon the digression. It has become fashionable in certain quarters of late, especially since the Spanish War and now that the coronation of his Majesty King Edward VII. is at hand, to affect an unusual friendship for the mother-country; and if it be only mutual and sincere I hope that this friendship may never grow less; but because we have taken our language and our common law from England, it does not follow, by any means, that we owe everything we have to England, and it does not follow that we must therefore ape England's antiquated customs, or that we are in any way dissatisfied with our own; and in this connection I am tempted to tell you another story I heard not long ago.

A gentleman travelling in this country and walking down Broadway one evening in the city of New York, feeling a little lonesome and inclined to be sociable, stopped a man to ask for a light, and, being accommodated, remarked, "This New York is quite a town. Came near getting lost several times to-day. A man doesn't seem to count for much here. Now, in London, don't you know, I was something. My name is Sir George Rawlins, Knight of the Garter, Knight of the Bath, Knight of the Golden Fleece, Knight of the——" and he went on to recite his many titles; and having finished the list he said, "And now, my man, pray what is your name?" "Oh, my name," said the man, who it seems was also a stranger in the country, and recently from the Emerald Isle, "my name is Michael Murphy, last night, the night before, the night before that, to-night, to-morrow night, and every night, just Michael Murphy."

But to return to our subject. The practitioner who through a want of professional skill or due care injures a patient is liable therefor under the common law in a suit for damages. This is now the law, always has been the law in Massachusetts, and, I venture to add, always will be, because founded on the unalterable decrees and principles of justice. But upon reflection, it will be easily seen that this did not meet the entire difficulty. A right of action was all very well, but few cared to adopt the remedy. The expense of litigation, the proverbial law's delay, the desire to avoid publicity, and very often the unaccountable indifference of individuals, especially in large communities, as to their own rights left the people an easy prey to every advertising, unscrupulous mountebank who might choose to enter and disgrace the profession. Anybody and everybody, with or without a college training or certificate of qualification, had a right to practise. Every college and dental school throughout the country, reputable and disreputable, had its own standard of qualification, or want of standard, and as to who were competent or incompetent the indiscriminating public had no means of knowing. Hence, the reason and necessity for further legislation. And to meet this condition of things the statute of 1887 was passed by our Legislature. This statute, with a few subsequent amendments, is now embodied in Chapter 76, Sections 24 to 29 inclusive, of the Revised Laws, which came into effect January 1, and have been lately published. With the provisions of this law the Society and members of the profession are of course familiar, and it needs no defence.

Every law to be just must be impartial, reasonable in its requirements, and for the good of the public; and this fairly describes our Massachusetts Dental Law. The controlling feature is that only those who show themselves to be duly qualified by a satisfactory examination before the State Board of Registration shall be permitted to practise. All else is detail.

The advantages of this law must be apparent. Chiefly, it establishes a central board of examiners who are appointed by the governor and directly responsible to the State; it fixes a high and a uniform standard of qualification throughout the State which no other system can provide, and if properly and judiciously enforced it secures to the public that reasonable measure of protection which it is the duty of the Commonwealth to furnish. Besides, the moral effect of such a law is not without its influence. It maintains the high standard of the profession and inspires the confidence of the public. The honest practitioner gives it his hearty approval and support. The laggard and the charlatan alone complain.

There is one other point to which I wish to call attention, and I have done. The vindication of every good law lies in its prosecution. Useless and in vain is that law which may be violated with impunity. A law that becomes a dead letter is worse than no law at all. Every law to be effective must be enforced. These are acknowledged truisms. Now, under the common law, as we have seen, those only who suffer injury are entitled to sue for damages, so that the prosecution of the common law in this particular must be left to them. Prosecutions under this statute law belong on the criminal side of the court and principally devolve upon the State. The Board of Registration, among other things, is charged with the important duty of regular examinations and the issuing of certificates to successful applicants, but as to who shall undertake the duty of prosecuting violations of the law the statute itself is silent, and accordingly this duty rests chiefly with the regular police authorities. Of course, the board cannot be indifferent to open and flagrant violations of the law, and in my opinion it is entirely competent for them, as a board or as individuals, to furnish the police authorities with such facts and evidence as may come to their knowledge. But this criminal prosecution is at best an unpleasant duty, and as the law makes no provision for the necessary expense and loss of time this work involves, farther than as I have indicated the board can scarcely be expected to go.

It is also entirely proper and competent, in my opinion, for this Society, as a whole or by its duly appointed committees in the various cities and towns, to take such action and give such assistance to the local police in this respect as the Society may from time to time think best and determine.

But after all is said and done under the law, much must be left to the individual conscience, the good influence of this Society, and public opinion. The charlatan who contrives to get into the fold and the reckless practitioner, however incompetent, who only thinks of the money there is in it, and just escapes the law, must eventually find their proper level and be relegated to that limbo of unenviable practice and standing in the community which their effrontery and their conduct so richly deserve.

Money is not all there is to live for. Good name in every honorable profession at least should count for something, and in this connection I would recommend for the serious consideration of the Society an amendment to the present statute law, to the effect that the conviction of any practitioner of a felony or lesser crime involving the breach of any professional trust or serious malpractice shall operate as a cancellation of his certificate and a bar to future practice. The profession can ill afford to lower or permit to be lowered that degree of professional skill, conscientious service, and high moral standing which the good name and honor of the profession itself require and the general public, upon which it depends, have a right to demand.

And now, Mr. President, after taking much more of your time than I at first intended, and let me again say appreciating the honor conferred upon me, I respectfully submit these remarks and few suggestions of mine for your candid and unreserved criticism and consideration, hoping that they may prove to be of some service to the Society and through the Society to the profession and community at large. And ladies and gentlemen of the Massachusetts Dental Society, I thank you for your patience and attention.

(To be continued.)

